# Treatment for sulfur mustard lung injuries; new therapeutic approaches from acute to chronic phase

**DOI:** 10.1186/2008-2231-20-27

**Published:** 2012-09-10

**Authors:** Zohreh Poursaleh, Ali Amini Harandi, Ensieh Vahedi, Mostafa Ghanei

**Affiliations:** 1 Chemical Injuries Research Center, Baqiyatallah University of medical sciences, Mollasadra Street, 19945–546, Tehran, Iran

**Keywords:** Sulfur mustard, Lung, Chronic, Acute, COPD, Treatment

## Abstract

**Objective:**

Sulfur mustard (SM) is one of the major potent chemical warfare and attractive weapons for terrorists. It has caused deaths to hundreds of thousands of victims in World War I and more recently during the Iran-Iraq war (1980–1988). It has ability to develop severe acute and chronic damage to the respiratory tract, eyes and skin. Understanding the acute and chronic biologic consequences of SM exposure may be quite essential for developing efficient prophylactic/therapeutic measures. One of the systems majorly affected by SM is the respiratory tract that numerous clinical studies have detailed processes of injury, diagnosis and treatments of lung. The low mortality rate has been contributed to high prevalence of victims and high lifetime morbidity burden. However, there are no curative modalities available in such patients. In this review, we collected and discussed the related articles on the preventive and therapeutic approaches to SM-induced respiratory injury and summarized what is currently known about the management and therapeutic strategies of acute and long-term consequences of SM lung injuries.

**Method:**

This review was done by reviewing all papers found by searching following key words sulfur mustard; lung; chronic; acute; COPD; treatment.

**Results:**

Mustard lung has an ongoing pathological process and is active disorder even years after exposure to SM. Different drug classes have been studied, nevertheless there are no curative modalities for mustard lung.

**Conclusion:**

Complementary studies on one hand regarding pharmacokinetic of drugs and molecular investigations are mandatory to obtain more effective treatments.

## Background

A biofunctional mustard agent i.e., sulfur mustard, bis(2-chloroethyl) sulfide (SM), was used in several conflicts since the first World War and has caused more than 80% of all documented chemical war gas casualties [1-3].

It is well known that chemical agents have been used against military personnel during conventional warfare, however, due to the increasing threat of terrorist activities, the focus has now broadened to encompass the threat posed to civilians. It maintains to be a major threat for use in battlefields and terrorist activities against either military and civilian targets [4]. It has been estimated that more than 100,000 Iranians were exposed to chemical warfare agents during the 8-year Iraq-Iran war, and around 50,000 mustard gas-affected individuals are suffering from of chronic respiratory, eye and/or dermatological complications [5]. Most victims (approximately 30,000) have been manifested different degree of long-term lung injuries [6]. Ever since, SM has been remained as a potent military and civilian threat [7], thus efficient prophylactic/therapeutic measures of acute and chronic pulmonary complications may be the most important challenge and intriguing goal in these setting. Most of acute lung injury survivors are confined to the upper respiratory tract (pharyngeal, palatal, tracheal lesions) and/or lower respiratory tract that had caused to fatal consequences due to airways complications [8]. Over many years, several surveys on long-term effects of SM, were published on lower respiratory tract system that revealed most frequent long-term respiratory effects were including bronchiolitis obliterans (BO), chronic bronchitis, bronchiectasis and hyper reactive airway disease [5,9].

Initially, medical groups supposed that they were encountered to a simple and known respiratory disease, so diagnostic and treatment lines were followed by the existing methods for well known respiratory illnesses like as asthma or chronic obstructive pulmonary disease (COPD). After a while, owing to failure of the conventional remedies, less of improving illnesses and resumption of crippling symptoms and signs gradually have revealed different aspects of these disorders. For example, it is has been established that smoking is the most important factor causing COPD and the adverse effects are either dependent to dose and duration of exposure. Fortunately, smoking cessation improves the accelerated decline in forced expiratory volume in one second (FEV1) in COPD [10]. In other words, even in severe COPD smoking cessation slows the accelerated rate of lung function decline and improves survival compared with continued smoking [11]. But featuring different scenarios, mustard lung injury was developed after single exposure and was independent of dosage and has progressive course. Because of the nature of SM-induced lung injuries, term of “*Disorder*” has been used instead of “*Disease*”. Ghanei and colleagues named this disorder as “mustard lung” for the first time to emphasis on specific character of this entity [12].

More previous studies showed that BO has been known to be the main pathological features of lung lesions in mustard lung on the basis of the radiological, histological, spirometric and laboratory findings [13]. Despite initial assumption, comprehensive investigations confirmed that these patients do from experience either pulmonary fibrosis or pulmonary emphysema [14-16].

According to the indicated differences in the pathogenesis of COPD and mustard lung, different therapeutic approaches have been considered for these patients [17]. Considering this fact whereas there is no cure for mustard lung injury, the main strategy of treatment is to prevent injuries by an effective protection and if occurred, prevention of disease progression rather than curing them or treating their symptoms. Consequently, we reviewed all modalities by considering preventive approaches; primary prevention (methods to avoid occurrence of disease), secondary prevention (methods to diagnose and treat extant disease in early stages before it causes significant morbidity) and tertiary prevention (methods to reduce negative impact of extant disease by restoring function and reducing disease-related complications) like as pulmonary rehabilitation.

This review could assist the clinicians and researchers to efficiently target their studies in the field of therapeutic approaches to SM-induced pulmonary injury.

## Methods

All published studies concerning treatment of respiratory effects of SM were included until 2012 February. Publications will be identified from the following electronic databases: Medline/Pubmed, Scopus, Google Scholar, Embase, ISI Web of Knowledge, Biological Abstracts and Chemical Abstracts. To include all of studies in electronic databases, keywords such as sulfur mustard; lung; chronic; acute; COPD; treatment were used. Subsequently, the therapeutic approaches to SM-induced pulmonary injury have been analyzed. In this study, efficient prophylactic/therapeutic measures are classified as acute and long-term SM-induced pulmonary effects and in three categories of primary, secondary and tertiary prevention.

### Acute SM-induced pulmonary effects

After minutes to several hours of exposure to SM, casualties experience tracheobronchitis with hoarseness, hacking cough, dyspnea, chest pressure, sore throat due to edema and erythema of the pharynx and bronchial tree [18,19]. During the next 12 hours, the main objective clinical findings develop with increasing cough, tachypnea, sinus pain [20,21]. Bronchiolar obstruction vs. bronchospasm can be anticipated due to pseudomembrane formation within the airways, sloughing of epithelium, and lower airway obstruction. After 24 to 48 hours, severe exposures cause to hemorrhagic pulmonary edema, secondary pneumonia, and respiratory failure due to necrosis with high mortality [20]. In spirometric findings, more obstructive patterns than restriction have been shown (53% vs. 2%) [19]. Generally, abnormal pulmonary function tests and particularly restrictive patterns tend to worsen over time [21].

### Medical management of acute toxic effects of SM

#### Primary prevention

Primary prevention is the prevention of the disease in susceptible individuals through promotion of healthcare and specific protection. The goal of primary prevention is to decrease the number of new cases (incidence) of a disorder. The ability to rapidly detect, identify and monitor chemical warfare agents is imperative for the efficient use of both military and civilian defence resources. This knowledge allows the severity and extent of a hazard to be assessed so that areas that are clean or contaminated can be identified. Furthermore, the information acquired by these systems provides advice to military commanders and first responders, regarding the donning of individual protective equipment, sampling, handling, analysis procedures and medical countermeasures. At this stage, early diagnosis of attack or exposure and decontamination play a major role [22]. After exposure to SM vapor, first-aid measures triage and medical treatment required as soon as possible. It must be ordered antidotal treatment with 1–500 mg sodium thiosulphate per kilogram body weight immediately [23-25].

In general treatment, control of pain and sedation of the severely intoxicated patients may be required. Also, supportive care of the patients is very important in their management. Willems and his colleague have reported that the application of extracorporeal detoxification procedures, such as haemoperfusion and haemodialysis, have no definitely therapeutic or clinical effect, because active mustard has not been identified in blood taken from victims. Also, these procedures are not safe and may facilitate coagulation disorders and systemic infections in such immunocompromised patients [23,26].

At acute phase, radiological evidence could be normal despite clinical signs e.g. bronchial rale, wheezing and crackels but it is the best time to start therapeutic interventions. Patients with acute SM lung injuries often receive bronchodilators, corticosteroids, immunosuppressive agents, antibiotics, mucolytics, oxygen therapy, and physiotherapy according to the involved complication(s) [22,27,28]. Combination a number of other drugs such as cysteine, sodium citrate, promethazine, heparin may be effective for the raising their protective activity against SM lung injuries. Also, Vitamin E could be used in combination with corticosteroids for protection against chemical-induced lung injury. [27-30]. But these different treatments still have no optimal effectiveness and also have known adverse effects in this stage of disease.

L-NAME & L-TC: L-nitroarginine methyl ester (L-NAME) and L-thiocitrullline (L-TC) are applied as two effective and protective drugs against the acute toxicity of SM. Maximal protective effects of L-NAME are delayed up to 3 h post-SM exposure that were dependent on the continued presence of L-NAME in the medium and were persistent up to 48 h after SM exposure [31,32]. Conversely, the protection conferred by L-TC against SM was persistent, and did not require the continued presence of L-TC after the initial SM lesion was expressed [33-35]. Some studies have been carried out for treatment of lung effects of SM-exposed patients *in vivo* and *in vitro* are summarized in Tables 1 and 2.

**Table 1:** Treatment of lung effects of SM-exposed patients *in vitro *

**Reference**	**Authors, Year, Country**	**Design of study/Intervention(s)**	**Participants**	**Result/s**
[27]	Wigenstam et al. 2009, Sweden	Dexamethasone liposome-encapsuled vitamin E	Mouse	Effective
[30]	Hoesel et al. 2008,USA	Alpha/gamma-tocopherol	Rat	Effective
		NAC + alpha/gamma-tocopherol		
[35]	Sawyer et al. 1998. Canada	l-thiocitrulline	Chick embryo	Effective
[31]	Sawyer 1998. Canada	L-NAME	Chick embryo	Effective
[36]	Paromov et al. 2008,USA	NAC	Intercellular macrophages	Effective
[37]	Atkins et al. 2000,USA	NAC	Endothelial cells	Effective
[38]	Hultén et al. 1998, Sweden	BHT	Alveolar macrophages	Effective
		NAC		
[39]	Anderson et al. 2000,USA	NIA	Rat	Ineffective
		NAC		Effective
[40]	Gao et al. 2010,USA	azithromycin, clarithromycin, erythromycin, roxithromycin	Alveolar macrophages	Effective
[41]	Guignabert et al. 2005. USA	Doxycycline	Guinea pigs	Effective
[42]	Van Helden et al. 2004, Netherlands	surfactant curosurf salbutamol	Guinea pigs	Effective
				More effective
[43]	Raza et al. 2006, USA	Doxycycline	Human lung epithelial cells	Effective
[44]	Anderson et al. 2009	Aprotinin	Rat	Effective
[45]	Yourick et al. 1992	NIA	Guinea pigs	Ineffective
[46]	Wilde and Upshall. 1991	Esters of cysteine	Rat	Effective
[47]	Zboril et al. 2012	zero-valent iron nanoparticles ferrate(VI)/(III) composite	Invitro	Effective
[48]	Boskabady et al. 2011	Nigella sativa	Guinea pigs	Effective
		Nigella sativa + dexamethasone		
[49]	O’Neill et al. 2010	AEOL 10150	Rat	Effective

*Niacinamide (NIA),N-acetyl cysteine (NAC), L-nitroarginine methyl ester (L-NAME), Aeolus(AEOL-10150) a small-molecule antioxidant analogous to the catalytic site of superoxide dismutase, Butylated hydroxytoluene (BHT).

**Table 2:** **Treatment of lung effects of SM-exposed patients *in vivo***

**Reference**	**Authors, Year, Country**	**Design of study/Intervention(s)**	**Participants**	**Result/s**
[20]	Ghanei et al. 2005, Iran	Group 1: Oral prednisolone	65 mustard gas-exposed chronic bronchitis patients (Group 1:26 & Group 2:39)	Effective
		Group 2: Intravenous Methylprednisolone		Effective
[50]	Ghanei et al. 2004, Iran	Clarithromycin + Acetylcysteine	mustard gas-exposed patients17	Effective
[51]	Ghanei et al. 2008, Iran	Group 1: NAC	144mustard gas-exposed patients with normal pulmonary function test	Effective
		Group 2: Placebo	(Group 1:72 & Group 2:72)	Ineffective
[52]	Shohrati et al. 2008, Iran	Group 1: NAC	144mustard gas-exposed patients with impaired pulmonary function test	Effective
		Group 2: Placebo	(Group 1:72 & Group 2:72)	Ineffective
[53]	Boskabady et al. 2008, Iran	Group 1: PC(20) salbutamol	22 exposed to chemical warfare (Group 1:11 & Group 2:15)	Effective
		Group 2: PC(35) salbutamol	15 normal control	Ineffective
[54]	Ghanei et al. 2007, Iran	Group 1: Fluticasone Propionate and Salmetrol	105 participants warfare (Group 1:52 & Group 2:53)	More effective
		Group 2: Beclomethasone and Salbutamol Inhaler		Less effective
[55]	Panahi et al. 2006, Iran	Group 1: Interferon Gamma-1b + Prednisolone	36 exposed to chemical warfare (Group 1:18 & Group 2:18)	More effective
		Group2: Prednisolone + Salbutamol+Beclomethasone		Less effective
				
[56]	Ghanei et al. 2010, Iran	Air: Oxygen	24mustard gas-exposed patients	Less effective
		Helium: Oxygen		More effective

*N-acetyl cysteine (NAC).

### Long-term SM-induced pulmonary adverse effects

After acute phases, respiratory problems are the greatest cause of long-term disability among people with combat exposure to mustard gas [17]. Long-term mustard lung injuries include chronic bronchitis (59%), tracheobronchial stenosis (24%) (ranging from diffuse involvement to isolated glottic or subglottic stenosis), asthma (11%), bronchiectasis (9%), airway narrowing due to scarring (10%), rarely honeycomb lung pathology as like fibrosis, and slightly increased incidence of lung cancer after a latency of 20 years and more [6,57].

Previous cohort studies have shown underlying pulmonary diseases such as resistant asthma and cigarette smoking can change the structure of the small airways and cause irreversible airflow obstruction. Thus they may be interfered with diagnostic and therapeutic processes of mustard lung by causing similar clinical features [23]. Conversely, comorbidity of resistant asthma and/or cigarette smoking with mustard lung may delay common therapeutic responses of mustard lung.

Several investigations have been evaluated long-term markers of SM toxicity 20 years after exposure. Pourfarzam and colleagues well assigned the association of the serum levels of IL-8, IL-6, C-reactive protein (CRP) and RF with long-term pulmonary involvement. It was concluded that serum levels of these inflammatory mediators probably do not play a major role in pathogenesis and persistence of pulmonary complications and could not reflect the degree of severity of pulmonary involvement following SM exposure [14,58]. Also, numerous studies have documented the theory of considering the oxidative–antioxidative imbalance and dependent markers of pulmonary inflammation in the pathophysiology of SM-induced pulmonary lesion. For example, Shohrati et al. showed Patients with moderate-to-severe SM-induced lung injuries had a tendency to show decreased level of glutathione and increased level of malondialdehyde (MDA) rather than those with mild injuries [59].

Latest data also show that mutations in the tumor suppressor gene, p53 may increase incidence of early-onset lung cancer in SM-exposed injury patients [57]. On the other hand, recently various studies have been described associated pathologic sequela of SM poisoning. Tracheobronchomalacia (TBM) and air trapping caused by affecting small and large airways are often observed in high-resolution computerized tomography (HRCT) scans of the chest of mustard gas exposed patients proving significant airway disease including constrictive (obliterative) bronchiolitis. However, the process is different from BO resulted from lung transplantation [14,60].

There are some data which support the role of oxidative stress and apoptosis as two prominent mechanisms that are involved in pathogenesis of mustard lung [13,61].

#### Secondary prevention

##### Treatment of long-term toxic effects

Secondary prevention focuses on identifying and treating people with established disease. In secondary prevention, the goal is to lower the rate of established cases of the disorder in the population (prevalence). It is relevant that the therapeutic response of long-term pulmonary complications due to exposure to SM is supposed to be different from one person to another due to various internal factors (i.e., healthy status, underlying disease, and genetic tendency) and external factors (i.e., toxicities, duration and frequency of exposure, co-exposure, emergent and follow-up medical care, smoking, and synergistic effect of occupational exposure).

##### Corticosteroids

Inhaled corticosteroids (ICS) are widely used in the treatment of patients with COPD but still their effectiveness in COPD remains controversial [20,62,63]. Inflammatory cells accumulation in the respiratory tract and production of inflammatory mediators can be correlated directly with altered lung function in SM-induced lung injury suggests that steroids depend on the extent of lung injury play a key role in treatment [64]. Furthermore, corticosteroids are extensively used to resolve exacerbation of respiratory symptoms in mustard lung. It should be considered that since mustard lung disorder is contributed to higher morbidities rather than mortalities, maintenance prescription of oral corticosteroids just may increases the related complications and has no effect on patients’ survival. Therefore, although ICS are significantly helpful in this setting, long term oral corticosteroids consumption should be warranted considered for severe cases [65].

However, this point is important with regard to different etiologies that lack active inflammation in these patients. The serum levels of these inflammatory mediators probably do not have any major role in pathogenesis and persistence of pulmonary complications and cannot reflect the degree of severity of pulmonary involvement following SM exposure [14]. Also, ineffectiveness of corticosteroids in airway reversibility in more than 50% of mustard injured cases [20] may imply the absence of active eosinophilic inflammation in these patients.

##### N acetyl-cysteine (NAC)

N acetyl-cysteine can be used as a protective agent that enhance glutathione-S-transferase (GSH) synthesis, as well as prevent oxidative activation of NF-кB inducing endothelial cell death and generate a local inflammatory reaction associated with the release of endothelial-derived cytokines [50,66,67]. Stav et al. have expressed a beneficial effect of NAC on physical performance probably due to a reduction in air trapping in stable, moderate-to-severe COPD [68].

To date it is identified that SM or its analogs reacts with nucleic acids, proteins, lipids and small molecular weight metabolites such as glutathione and lead to oxidative stress. It can increase oxidative products include reactive oxygen species (ROS), lipid peroxidation products and oxidized proteins, and inhibits antioxidant enzymes such as superoxide dismutase, catalase, GSH, and thioredoxin reductase. This inequity by SM can damage pulmonary matrix cells and disrupt cellular redox homeostasis [29,36,37]. Mehrani et al. was also showed that S100 calcium-binding protein A8 and Apo A1 both increased in BAL fluid in endotoxin-challenged in patients with high severity of SM exposure [69]. Numerous investigations have suggested an anti-oxidant effect for these two proteins [70,71], which confirm the oxidative–antioxidative imbalance in the pathophysiology of SM-induced pulmonary lesion.

As a result, successful therapy for SM toxicity may depend on the development of new antioxidants effective against SM-induced ROS and their improved delivery to target tissues. Also, a previous study showed that SM causes a transient decrease in inducible nitric oxide synthase (iNOS) protein syntheses rather than a direct inhibition of iNOS activity due to covalent modifications by SM [72]. In such setting where oxidative stress is an ongoing and long term process, using antioxidative modalities should be started soon in early stage of disease to maintain oxidant-antioxidant balance and continued as long as the underlying pathology is persisting.

The NAC would prevent GSH depletion and restore the loss of iNOS activity in stimulated macrophages and in rat model it could protect bronchial epithelial cells against SM *in vitro*[36,37]. In addition, NAC can reduce the level of tumor necrosis factor-α (TNFα) in lung transplanted persons [38,73]. It also reduces the secretion of many inflammatory modulators and could prevent thickening of respiratory airways and bronchial smooth muscle hypertrophy. Therefore NAC not only can be used in treating bronchitis and bronchiolitis, but also prevent mustard induced pulmonary lesions due to oxidative stress. An *in vivo* study has been showed NAC vs. niacinamide (NIA) has more effective against SM-induced lung injury that determined by biomedical and cytological analysis of lavage fluid [39]. In a double-blind clinical trial study on 144 SM-induced BO patients with normal PFT improved dyspnea, wake-up dyspnea and cough after 4 months of NAC administration compared with the placebo group. NAC reduced sputum from 76.9% of cases before the trial to 9.6% of cases after the trial. Spirometric components were significantly improved in NAC group compared with the placebo group [51]. Also, in another double-blind clinical trial study on mustard gas-exposed patients with impaired pulmonary function test, clinical-conditions and spirometric findings in these patients can improve after fourth months administration of NAC [52].

Some different studies have been carried out based on antioxidant properties for treatment of respiratory adverse effects of SM-exposed patients *in vivo* and *in vitro*.

##### Bronchodilators

Bronchodilators can be applied in patients with increased airway hyper-reactivity and especially for moderate to severe cases with SM exposed lung injuries [72]. Combination of β-agonist (e.g., salbutamol) and an anticholinergic (e.g., ipratropium bromide) has been found to be more effective than any of other bronchodilators used alone in these patients [53]. A study has shown combination therapy with long-acting beta 2-agonists and inhaled corticosteroids for 12 weeks. In addition, short-term intravenous pulse or oral corticosteroid therapy can improve FEV1, FVC and PEF of patients with mustard gas-induced chronic bronchitis in the case of exacerbation, and this effect is synergistic with inhaled β -2 agonist bronchodilators [54].

##### Macrolides

It has been proven that macrolide antibiotics may serve as potential vesicant respiratory therapeutics. Macrolides are effective in reducing SM-induced overproduction of proinflammatory cytokines and mediators, as well as improving the degenerated chemotactic and phagocytotic functions of monocytes following SM exposure. Also, macrolide antibiotics may lead to clearance improvement of apoptotic material in the airway and ultimately cause to reduce airway inflammation due to SM inhalation [40,74]. Specifically, mustard lung patients are observed to have severe and persistent respiratory symptoms in the absence of eosinophilic inflammation. In other word non-eosinophilic (neutrophil mediated) inflammation is relatively common. This would have major consequences for the treatment and prevention since most previous treatment and prevention strategies were almost entirely focused on allergic and eosinophilic measures. In such a non-eosinophilic inflammation the macrolides are one of best candidate to play their anti-inflammatory role.

We should emphasize that previous studies showed to excess neutrophil in BAL studies [15], to decrease the circulating levels of IL-6, IL-8, CPR, RF in SM exposed patients with various degrees of late pulmonary complications [13,14] and to increase expression of markers of inflammation including cyclooxygenase-2 (COX-2), TNFα, iNOS, matrix metalloproteinase-9 (MMP-9) and a significant increase in total protein, IL-1∝ and IL-13 that each of which has been implicated in pulmonary toxicity on SM exposed model [16,25]. But, other surveys have been noted that there is no active inflammation in these patients because corticosteroids are ineffectiveness in airway reversibility in more than 50% of mustard injured cases [20]. In other words, the efficacy of inflammation mechanisms is really unobvious in pathogenesis pulmonary complications and does not reflect the degree of severity of pulmonary involvement following SM exposure [14].

#### Evaluation and treatment of gastroesophageal reflux disease (GERD)

Studies of patients with chronic upper airway symptoms describe a potential relationship between GERD and upper airway conditions. The hypothetical mechanisms linking upper respiratory symptoms with GERD include heightened bronchial reactivity, microaspiration, and a vagally mediated reflex mechanism [75]. However, in the case of GERD-related respiratory symptoms the typical digestive syndrome is frequently absent, the situation corresponding to the so-called ‘silent GERD’ [76]. The relationship of upper respiratory symptoms and GERD is based upon investigations of subjects with persistent upper respiratory symptoms. In the majority of these cases, GERD was asymptomatic or the symptoms were not sufficiently severe to warrant an evaluation of GERD before the evaluation of upper respiratory symptoms [77]. Treatment of GERD could result in marked improvement or even disappearance of symptoms in patients with chronic respiratory symptoms. In addition in the specific situation of GERD-related respiratory problems, empirical therapy with proton pomp inhibitors (PPI) is frequently proposed in these cases [76].

Some studies addressed the correlation between respiratory symptoms in mustard lung disorder and GERD symptoms [77]. In a study by evaluating of chronic cough in mustard lung patients GERD was accounted for 44% of chronic cough of the patients [78]. Evaluation of GERD and treatment or even empirical therapy with a PPI especially in symptomatic and uncontrolled respiratory symptoms is highly recommended in mustard lung to relief of respiratory symptoms associated with GERD.

##### Protease inhibitors

There is some evidence for the role for proteases such as serine, cysteine, and MMP in the pathogenesis of SM-induced damage, even in the SM exposed model [21,44]. SM inhalation may cause cell death of airway macrophages by themselves, as well as degenerated chemotactic and phagocytic functions of the surviving cells. As a result, as hallmarks of SM inhalation exposure, these consequences may affect incompetent clearance of dead cells, and accumulation of apoptotic and necrotic fragments promoting the ongoing inflammation and losing of structural integrity in the airway. These processes were caused to damage normal pulmonary parenchyma and regard to emphysema. Absence of emphysema in nonsmoker SM-induced lung injury is against over activity proteolytic molecules in these patients.

Previous studies reported treatment with aprotinin and to a lesser extent ilomastat, as well as doxycycline reduce some of the direct inflammatory response and damage associated with SM-induced lung injury [79]. Aprotinin irreversibly binds to proteases that contain a serine amino acid residue in their catalytic site inhibiting the actions of trypsin, plasmin, kallikrien, elastase, urokinase, and thrombin in an increasing dose-dependent manner, respectively [80].

Doxycycline also exhibits non-specific MMP inhibitory activity such as MMP-2, MMP-9 and cellularity and protein levels. In addition, doxycycline and related tetracyclines can be influence on iNOS expression and nitric oxide production, to reduce inflammatory cytokine release, and to scavenge reactive oxygen species [41,43,65].

##### ∝1-antitrypsin activity (AAT)

Previous studies showed that AAT due to oxidative stress has been diminished after exposure to SM. But significant difference in PI phenotype and the effect of SM on the mutation of the AAT gene is unlikely. Additionally, it could be concluded that just a single exposure to SM has no effect on changing the phenotype of AAT [81]. AAT deficiency has no cure. Thus, people who have AAT deficiency and moderate to severe impairment of pulmonary function due to long time exposure to SM could be continued medical care and lifestyle changes that help to manage their health. Medical care may included inhaled bronchodilators and inhaled steroids, protection with Flu and pneumococcus vaccines, pulmonary rehabilitation, and if needed, extra oxygen therapy.

##### Interferon gamma-1b (INF γ-1b)

It has been recently demonstrated that transforming growth factor *β*1 (TGF-b1) target protein and plays a fundamental role in BO pathogenesis and is substantially increased in BAL aspirates and target tissues of SM exposed patients, compared with non-exposed individuals. Also, TGF-β especially TGF-β1 increase in SM- exposed patients. This factor has been described to play an important role in the pathogenesis of progressive inflammatory and fibrotic diseases such as IPF and BO [17]. Ghanei et al., reported that honey combing that is typical for pulmonary fibrosis is not found in lung mustard and BO is main complication of these patients [7]. In addition, current treatments of BO in post-lung transplant patients i.e., immunosuppression and corticosteroid therapy, are not effective in SM exposed patients [18,19]. The IFN-γ1b is a bioengineered form of interferon gamma, a protein that acts as a biologic response modifier through stimulation of the human immune system. We presume that the response to treatment in our patients can be attributed to the down regulating effects of IFN-γ1b on TGF-b1 [17,55,82]. Recently, it was shown that six-month treatment with IFN-γ1b plus a low-dose prednisolone could improve pulmonary function of SM-exposed patients with severe delayed lung complications [55,82].

##### Surfactant therapy

Recent studies on animal model have also proposed surfactants in combination with bronchodilators or anti-inflammatory agents. This medication may be useful in SM induced lung injury [16,42].

##### Angiotensin-converting enzyme (ACE) genotype

ACE genotype and the Renin Angiotensin system influence the severity of the late respiratory complications of mustard gas exposure with the D allele being associated higher FEV 1% predicted. It is evidence that ACE influences may open the way to new therapeutic options [83].

#### Treatment during exacerbation

In a recent report has suggested that the use of heliume oxygen mixtures with non-invasive ventilation can be decreased airway resistance and work of breathing in subjects with chronic dyspnea following SM exposure. Therefore, helium oxygen mixtures usage may improve impairment of function during exacerbation and also pulmonary in moderate to severe chronic lung effects [56].

Other therapies during exacerbation are short courses of systemic corticosteroids or inhaled corticosteroids, antibiotics, morphine, oxygen supplement therapy, mucolytics and chest physiotherapy. However, the optimal choice of antibiotic and length of use are still unclear.

#### Tertiary prevention and rehabilitation

Tertiary prevention commonly includes the prevention of the disease progression after it is clinically noticeable and the diagnosis has been established. In fact it seeks to stabilize or decrease the amount of disability associated with an existing disorder. It was shown that after long term follow up that mortality is usually low in patients with injuries of SM [84]. It leads to high prevalence of victims and frequent end stage morbidities. In such cases exercise rehabilitation shows significant results. Also long Long-term supplemental oxygen therapy and nasal intermittent positive pressure ventilation have been prescribed.

It is of note that lung transplantation because they have a long term survival. In addition there is no evidence regarding possible effect(s) of underlying lung pathology on post transplant lung tissue.

##### New therapeutic approach

First, it is very important to note that regarding to the pathogenesis of the disorder (oxidant-antioxidant imbalance) in patients with injuries of SM, we expect to patients response to the treatment with anti oxidative agents [59]. Hence, every therapeutic agent that can enhance cellular anti-oxidant supply may be effective in such these patients. In our studies that not published yet, we have been assessing new therapeutic approach as Curcumin, Hypertonic saline and Manitol in SM injured patients.

##### Curcumin

There are so many evidences which confirmed the efficacy of Curcumin as a herbal medication on inflammation, antioxidant supply, neoplasm and on the apoptotic pathway (such as NF-KB) [85,86]. Curcumin can affect the TGF-beta/Smads signaling pathway and also can regulate inter-cellular adhering molecule (such as Laminin and cathepsin that was presented as one of the main mechanisms of SM injury [87-89]. There is some evidence that is confirmed the efficacy of Curcumin on the response of airway epithelial cell to toxic agents [90]. Therefore, it seems that it can be effective on control of severity of SM injury disorder although the main problem in using this drug was the low systemic availability because of the efficient first-pass metabolism and some degree of intestinal metabolism when administered via the oral [91]. If the efficient Liposomal formula of Curcumin is produced can impressively use as inhaled, oral or intra venous in SM injured.

##### Hypertonic saline (HTS)

It is a sodium chloride solution with hyper osmolarity which used for septic shock, bronchiolitis and sputum induction [92]. Nebulization with HTS can regulate sodium transport across airway lining [93]. Inhalation of HTS improve cilliary function of epithelial cell in which can be useful in these patients [94]. Nowadays, the effect of HTS on the inflammatory cytokines such as IL-8 was demonstrated [12,95]. Nebulization of HTS 5% was safely used [96] and can be superior to current therapy for early treatment of SM injured patient with Bronchiolitis obliterans.

##### Mannitol

Inhaled Mannitol have several effect on lung like HTS such as regulating water flows into the airway lumen via osmotic gradient modification and diluting airways mucus and improving muco-ciliary function and therefore could have a role in treating chronic suppurative lung disease and resolution of airway inflammation such as SM injured lung [97,98]. On the other hand, its formulation as inhaler or nebulizer makes it easy to use but headache as the most common adverse effect limited the interest to use it.

## Conclusion

Mustard lung has an ongoing pathological process and is an active disorder even years after exposure to SM. Although the previous researches were effectively used biological or non-biological medications for SM injuried lung [45-49], there are no curative modalities for mustard lung. Therefore the main goal is primary prevention and if injury had been occurred, secondary prevention for victim should be considered. In disabled and end stage victims some tertiary preventive method are available. Complementary studies on one hand regarding pharmacokinetic of drugs and molecular investigations to understand more about underlying physiopathology of such a disorder, and on the other hand performing more survey and controlled clinical trials are mandatory to obtain more effective treatments

## Competing interests

The authors declare that they have no competing interests.

## Authors’ contribution

MG corresponded, conception and design and final revision; ZP acquisition of data, analysis and interpretation of data, drafting the article, revising it critically for important intellectual content, writing first draft of manuscript; AAH acquisition of data, revising it critically for important intellectual content. EV revising it critically for important intellectual content. All authors read and approved the final manuscript.

## Authors information

Chemical Injuries Research Center, Baqiyatallah University of medical sciences, Mollasadra Street, 19945‐546, Tehran, Iran.

## References

[B1] Ghabili K Agutter PS Ghanei M Ansarin K Shoja MM Mustard gas toxicity: the acute and chronic pathological effects J Appl Toxicol 2010 30 7 627 643 10.1002/jat.1581 20836142

[B2] Sidell FR Urbanetti JS Smith WJ Zajtchuk R, Bellamy RF Vesicants Medical Aspects of Chemical and Biological Warfare 1997 Washington, DC: Office of the Surgeon General-Department of the Army; United States of America 197 228

[B3] Prentiss AM Vesicant agents Chemicals in warfare: a treatise on chemical warfare 1937 New York: USA 177 300

[B4] Saladi RN Smith E Persaud AN Mustard: A potential agent of chemical warfare and terrorism Clin Exp Dermatol 2006 31 1 5 10.1111/j.1365-2230.2005.01945.x 16309468

[B5] Emad A Rezaian GR The diversity of the effects of sulfur mustard gas inhalation on respiratory system 10 years after a single, heavy exposure: analysis of 197 cases Chest 1997 112 3 734 738 10.1378/chest.112.3.734 9315808

[B6] Foroutan SA Medical Notes on the Chemical Warfare: Part II (in persion) Kowsar Med J 1997 1 159 177

[B7] Ghanei M Mokhtari M Mohammad MM Aslani J Bronchiolitis obliterans following exposure to sulfur mustard: chest high resolution computed tomography Eur J Radiol 2004 52 2 164 169 10.1016/j.ejrad.2004.03.018 15489074

[B8] Urbanetti JS Loke J Battlefield chemical inhalation injury Pathophysiology and Treatment of Inhalation Injuries 1988 New York: Marcel Dekker, Inc 281 348

[B9] Thomason JW Rice TW Milstone AP Bronchiolitis obliterans in a survivor of a chemical weapons attack JAMA 2003 290 5 598 599 10.1001/jama.290.5.598 12902361

[B10] Willemse BW Postma DS Timens W ten Hacken NH The impact of smoking cessation on respiratory symptoms, lung function, airway hyperresponsiveness and inflammation Eur Respir J 2004 23 3 464 476 10.1183/09031936.04.00012704 15065840

[B11] Godtfredsen NS Lam TH Hansel TT Leon ME Gray N Dresler C COPD-related morbidity and mortality after smoking cessation: status of the evidence Eur Respir J 2008 32 4 844 853 10.1183/09031936.00160007 18827152

[B12] Ghanei M Harandi AA Molecular and cellular mechanism of lung injuries due to exposure to sulfur mustard: a review Inhal Toxicol 2011 23 7 363 371 10.3109/08958378.2011.576278 21639706 PMC3128827

[B13] Attaran D Lari SM Khajehdaluee M Ayatollahi H Towhidi M Asnaashari A Highly sensitive C-reactive protein levels in Iranian patients with pulmonary complication of sulfur mustard poisoning and its correlation with severity of airway diseases Hum Exp Toxicol 2009 28 12 739 745 10.1177/0960327109354311 19919970

[B14] Ghanei M Harandi AA Long term consequences from exposure to sulfur mustard: a review Inhal Toxicol 2007 19 451 456 10.1080/08958370601174990 17365048

[B15] Mehrani H Ghanei M Aslani J Golmanesh L Bronchoalveolar lavage fluid proteomic patterns of sulfur mustard-exposed patients Proteomics Clin Appl 2009 3 1191 1200 10.1002/prca.200900001 21136943

[B16] Malaviya R Sunil VR Cervelli J Anderson DR Holmes WW Conti ML Inflammatory effects of inhaled sulfur mustard in rat lung Toxicol Appl Pharmacol 2010 248 2 89 99 10.1016/j.taap.2010.07.018 20659490 PMC3954123

[B17] Aghanouri R Ghanei M Aslani J Keivani-Amine H Rastegar F Karkhaneh A Fibrogenic cytokine levels in bronchoalveolar lavage aspirates 15 years after exposure to sulfur mustard Am J Physiol Lung Cell Mol Physiol 2004 287 1160 1164 10.1152/ajplung.00169.2003 15286001

[B18] Laohaburanakit P Chan A Allen RP Bronchiolitis obliterans Clin Rev Allergy Immunol 2003 25 3 259 274 10.1385/CRIAI:25:3:259 14716071

[B19] Chan A Allen R Bronchiolitis obliterans: an update Curr Opin Pulm Med 2004 10 2 133 141 10.1097/00063198-200403000-00008 15021183

[B20] Ghanei M Khalili AR Arab MJ Mojtahedzadeh M Aslani J Lessan-Pezeshki M Diagnostic and therapeutic value of short-term corticosteroid therapy in exacerbation of mustard gas-induced chronic bronchitis Basic Clin Pharmacol Toxicol 2005 97 5 302 305 10.1111/j.1742-7843.2005.pto_187.x 16236142

[B21] Cowan FM Anderson DR Broomfield CA Byers SL Smith WJ Biochemical alterations in rat lung lavage fluid following acute sulfur mustard inhalation:II. Increase in proteolytic activity Inhal Toxicol 1997 9 53 61 10.1080/089583797198411

[B22] Sferopoulos R A Review of Chemical Warfare Agent (CWA) Detector Technologies and Commercial-Off-The-Shelf Items 2009 DSTO-GD-0570

[B23] Balali-Mood M Hefazi M The pharmacology, toxicology, and medical treatment of sulphur mustard poisoning Fundam Clin Pharmacol 2005 19 3 297 315 10.1111/j.1472-8206.2005.00325.x 15910653

[B24] Zhang Z Riviere JE Monteiro-Riviere NA Evaluation of protective effects of sodium thiosulfate, cysteine, niacinamide and indomethacin on sulfur mustard-treated isolated perfused porcine skin Chem Biol Interact 1995 96 3 249 262 10.1016/0009-2797(94)03596-Z 7750164

[B25] Shakarjian MP Bhatt P Gordon MK Chang YC Casbohm SL Rudge TL Preferential expression of matrix metalloproteinase-9 in mouse skin after sulfur mustard exposure J Appl Toxicol 2006 26 3 239 246 10.1002/jat.1134 16489579

[B26] Willems JL Clinical management of mustard gas casualties Ann Med Mil Belg 1989 3 1 61

[B27] Wigenstam E Rocksén D Ekstrand-Hammarström B Bucht A Treatment with dexamethasone or liposome-encapsuled vitamin E provides beneficial effects after chemical-induced lung injury Inhal Toxicol 2009 21 11 958 964 10.1080/08958370802596298 19572781

[B28] Vojvodić V Milosavljević Z Bosković B Bojanić N The protective effect of different drugs in rats poisoned by sulfur and nitrogen mustards Fundam Appl Toxicol 1985 5 6 Pt 2 S160 S168 4092884

[B29] Laskin JD Black AT Jan Y Sinko PJ Heindel ND Sunil V Oxidants and antioxidants in sulfur mustard–induced injury Ann N Y Acad Sci 2010 1203 92 100 10.1111/j.1749-6632.2010.05605.x 20716289 PMC4023473

[B30] Hoesel LM Flierl MA Niederbichler AD Rittirsch D McClintock SD Reuben JS Ability of antioxidant liposomes to prevent acute and progressive pulmonary injury Antioxid Redox Signal 2008 10 5 973 981 18257742 10.1089/ars.2007.1878

[B31] Sawyer TW Characterization of the protective effects of L-nitroarginine methyl ester (L-NAME) against the toxicity of sulphur mustard in vitro Toxicology 1998 13 1 21 32 9881932 10.1016/s0300-483x(98)00120-6

[B32] Callaway S Pearce KA Protection against systemic poisoning by mustard gas, di(2-chloroethyl) sulphide, by sodium thiosulphate and thiocit in the albino rat Br J Pharmacol 1958 13 13 395 398 10.1111/j.1476-5381.1958.tb00227.x PMC1481880 13618542

[B33] Foster JH Lewis MR Jacobs JK Thiosulfate protection against the toxic effects of nitrogen mustard in perfusion of the liver Am J Surg 1962 28 461 464 13894237

[B34] Connors TA Protection against the toxicity of all alkylating agents by thiols: the mechanism of protection and its relevance to cancer chemotherapy Eur J Cancer 1966 2 4 293 305 5342910 10.1016/0014-2964(66)90042-9

[B35] Sawyer TW Hancock JR D’Agostino PA l-thiocitrulline: A potent protective agent against the toxicity of sulphur mustard in vitro Toxicol Appl Pharmacol 1998 151 2 340 346 10.1006/taap.1998.8457 9707510

[B36] Paromov V Qui M Yang H Smith M Stone WL The influence of N-acetyl-L-cysteine on oxidative stress and nitric oxide synthesis in stimulated macrophages treated with a mustard gas analogue BMC Cell Biol 2008 9 33 10.1186/1471-2121-9-33 18570648 PMC2446388

[B37] Atkins KB Lodhi IJ Hurley LL Hinshaw DB N-Acetylcysteine and Endothelial Cell Injury by Sulfur Mustard J Appl Toxicol 2000 20 1 125 128 10.1002/1099-1263(200012)20:1+<::aid-jat671>3.0.co;2-u 11428622

[B38] Hultén LM Lindmark H Scherstén H Wiklund O Nilsson FN Riise GC Butylated hydroxytoluene and N-acetylcysteine attenuates tumor necrosis factor-alpha (TNF-alpha) secretion and TNF-alpha mRNA expression in alveolar macrophages from human lung transplant recipients in vitro Transplantation 1998 66 3 364 369 15 10.1097/00007890-199808150-00014 9721806

[B39] Anderson DR Byers SL Vesely KR Treatment of sulfur mustard (HD)-induced lung injury J Appl Toxicol 2000 20 Suppl 1 S129 S132 11428623 10.1002/1099-1263(200012)20:1+<::aid-jat670>3.0.co;2-x

[B40] Gao X Ray R Xiao Y Ishida K Ray P Macrolide antibiotics improve chemotactic and phagocytic capacity as well as reduce inflammation in sulfur mustard-exposed monocytes Pulm Pharmacol Ther 2010 23 2 97 106 10.1016/j.pupt.2009.10.010 19895898

[B41] Guignabert C Taysee L Calvet JH Planus E Delamanche S Galiacy S d’Ortho MP Effect of doxycycline on sulfur mustard-induced respiratory lesions in guinea pigs Am J Physiol Cell Mol Physiol 2005 289 L67 L74 10.1152/ajplung.00475.2004 15778244

[B42] Van Helden HP Kuijpers WC Diemel RV Asthma like symptoms following intratracheal exposure of Guinea pigs to sulfur mustard aerosol: therapeutic efficacy of exogenous lung surfactant curosurf and salbutamol Inhal Toxicol 2004 16 537e48 15204745 10.1080/08958370490442520

[B43] Raza M Ballering JG Hayden JM Robbins RA Hoyt JC Doxycycline decreases monocyte chemoattractant protein-1 in human lung epithelial cells Exp Lung Res 2006 32 15e26 16809218 10.1080/01902140600691399

[B44] Anderson DR Taylor SL Fetterer DP Holmes WW Evaluation of protease inhibitors and an antioxidant for treatment of sulfur mustard-induced toxic lung injury Toxicology 2009 263 41e6 18852015 10.1016/j.tox.2008.08.025

[B45] Yourick JJ Dawson JS Mitcheltree LW Sulfur mustard-induced microvesication in hairless guinea pigs: effect of short-term niacinamide administration Toxicol Appl Pharmacol 1992 117 1 104 109 10.1016/0041-008X(92)90223-F 1440603

[B46] Wilde PE Upshall DG Cysteine esters protect cultured rodent lung slices from sulphur mustard Hum Exp Toxicol 1994 13 11 743 748 10.1177/096032719401301102 7857693

[B47] Zboril R Andrle M Oplustil F Machala L Tucek J Filip J Marusak Z Sharma VK Treatment of chemical warfare agents by zero-valent iron nanoparticles and ferrate(VI)/(III) composite J Hazard Mater 2012 211-212 126 130 22119195 10.1016/j.jhazmat.2011.10.094

[B48] Boskabady MH Vahedi N Amery S Khakzad MR The effect of Nigella sativa alone, and in combination with dexamethasone, on tracheal muscle responsiveness and lung inflammation in sulfur mustard exposed guinea pigs J Ethnopharmacol 2011 37 2 1028 1034 21801826 10.1016/j.jep.2011.07.030

[B49] O’Neill HC White CW Veress LA Hendry-Hofer TB Loader JE Min E Treatment with the catalytic metalloporphyrin AEOL 10150 reduces inflammation and oxidative stress due to inhalation of the sulfur mustard analog 2-chloroethyl ethyl sulfide Free Radic Biol Med 2010 48 9 1188 1196 10.1016/j.freeradbiomed.2010.01.039 20138141 PMC2847650

[B50] Ghanei M Abolmaali K Aslani J Efficacy of concomitant administration of clarithromycin and acetylcysteine in bronchiolitis obliterans in seventeen sulfur mustard exposed patients: An open-label study Curr Ther Res 2004 6 495 504 10.1016/j.curtheres.2004.12.001 PMC3964536 24672101

[B51] Ghanei M Shohrati M Jafari M Ghaderi S Alaeddini F Aslani J N-acetylcysteine improves the clinical conditions of mustard gas-exposed patients with normal pulmonary function test Basic Clin Pharmacol Toxicol 2008 103 5 428 432 10.1111/j.1742-7843.2008.00318.x 18801028

[B52] Shohrati M Aslani J Eshraghi M Alaedini F Ghanei M Therapeutics effect of N-acetyl cysteine on mustard gas exposed patients: evaluating clinical aspect in patients with impaired pulmonary function test Respir Med 2008 102 443 448 10.1016/j.rmed.2007.10.004 18036807

[B53] Boskabady MH Attaran D Shaffei MN Airway responses to salbutamol after exposure to chemical warfare Respirology 2008 13 2 288 293 10.1111/j.1440-1843.2007.01157.x 18339031

[B54] Ghanei M Shohrati M Harandi AA Eshraghi M Aslani J Alaeddini F Inhaled corticosteroids and long-acting beta 2-agonists in treatment of patients with chronic bronchiolitis following exposure to sulfur mustard Inhal Toxicol 2007 19 10 889 894 10.1080/08958370701432132 17687719

[B55] Panahi Y Ghanei M Aslani J Mojtahedzadeh M The therapeutic effect of gamma interferon in chronic bronchiolitis due to mustard gas Iran J Allergy Asthma Immunol 2005 4 2 83 90 17301427

[B56] Ghanei M Rajaeinejad M Motiei-Langroudi R Alaeddini F Aslani J Helium:oxygen versus air:oxygen noninvasive positive-pressure ventilation in patients exposed to sulfur mustard Heart Lung 2011 40 3 e84 e89 10.1016/j.hrtlng.2010.04.001 20561871

[B57] Hosseini-khalili A Haines DD Modirian E Soroush M Khateri S Joshi R Zendehdel K Ghanei M Giardina C Mustard gas exposure and carcinogenesis of lung Mutat Res 2009 678 1 1 6 10.1016/j.mrgentox.2009.05.022 19559099 PMC2811761

[B58] Pourfarzam S Ghazanfari T Yaraee R Ghasemi H Hassan ZM Faghihzadeh S Serum levels of IL-8 and IL-6 in the long term pulmonary complications induced by sulfur mustard: Sardasht-Iran Cohort Study Int Immunopharmacol 2009 9 13–14 1482 1488 19748599 10.1016/j.intimp.2009.09.002

[B59] Shohrati M Ghanei M Shamspour N Babaei F Abadi MN Jafari M Glutathione and malondialdehyde levels in late pulmonary complications of sulfur mustard intoxication Lung 2010 188 1 77 83 10.1007/s00408-009-9178-y 19862574

[B60] Ghanei M Akbari-Moqadam F Mir-Mohammad M Aslani J Tracheobronchomalacia and air trapping after mustard gas exposure Am J Respir Crit Care Med 2006 173 3 304 309 10.1164/rccm.200502-247OC 16254272

[B61] Hodge SJ Hodge GL Reynolds PN Scicchitano R Holmes M Increased production of TGF-beta and apoptosis of T lymphocytes isolated from peripheral blood in COPD Am J Physiol Lung Cell Mol Physiol 2003 285 492 499 10.1152/ajplung.00428.2002 12851215

[B62] Ghanei M Hosseini AR Arabbaferani Z Shahkarami E Evaluation of chronic cough in chemical chronic bronchitis patients Environ Toxicol Pharmacol 2005 20 1 6 10 10.1016/j.etap.2004.09.006 21783560

[B63] Niewoehner DE Erbland ML Deupree RH Collins D Gross NJ Light RW Effect of systemic glucocorticoids on exacerbations of chronic obstructive pulmonary disease N Engl J Med 1999 340 25 1941 1947 10.1056/NEJM199906243402502 10379017

[B64] Yaraee R Ghazanfari T Ebtekar M Ardestani SK Rezaei A Kariminia A Alterations in serum levels of inflammatory cytokines (TNF, IL-1alpha, IL-1beta and IL-1Ra) 20 years after sulfur mustard exposure: Sardasht-Iran cohort study Int Immunopharmacol 2009 9 13–14 1466 1470 19747989 10.1016/j.intimp.2009.09.001

[B65] Culpitt SV Maziak W Loukidis S Nightingale JA Matthews JL Barnes PJ Effect of high dose inhaled steroid on cells, cytokines, and proteases in induced sputum in chronic obstructive pulmonary disease Am J Respir Crit Care Med 1999 160 1635 1639 10556133 10.1164/ajrccm.160.5.9811058

[B66] Dekhuijzen PN Acetylcysteine in the treatment of severe COPD Ned Tijdschr Geneeskd 2006 150 22 1222 1226 3 16796172

[B67] Soltan-Sharifi MS Mojtahedzadeh M Najafi A Reza Khajavi M Reza Rouini M Moradi M Improvement by N-acetylcysteine of acute respiratory distress syndrome through increasing intracellular glutathione, and extracellular thiol molecules and anti-oxidant power: evidence for underlying toxicological mechanisms Hum Exp Toxicol 2007 26 9 697 703 10.1177/0960327107083452 17984140

[B68] Stav D Raz M Effect of N-acetylcysteine on air trapping in COPD: a randomized placebo-controlled study Chest 2009 136 2 381 386 10.1378/chest.09-0421 19447919

[B69] Mehrani H Ghanei M Aslani J Tabatabaei Z Plasma proteomic profile of sulfur mustard exposed lung diseases patients using 2-dimensional gel electrophoresis Clin Proteomics 2011 8 1 2 21906349 10.1186/1559-0275-8-2 PMC3167199

[B70] Passey RJ Xu K Hume DA Geczy CL S100 A8: emerging functions and regulation J Leukoc Biol 1999 66 549 556 10534107 10.1002/jlb.66.4.549

[B71] Gabay C Kushner I Acute-phase proteins and other systemic responses to inflammation N Engl J Med 1999 340 448 454 10.1056/NEJM199902113400607 9971870

[B72] Aslani J Cheraghali AM Late respiratory complications of sulphur mustard Prevention and treatment of complications of chemical warfare agents 2000 Tehran, Iran: Chemical Warfare Research Centre 76 79

[B73] Bergmann M Tiroke A Schäfer H Barth J Haverich A Gene expression of profibrotic mediators in bronchiolitis obliterans syndrome after lung transplantation Scand Cardiovasc J 1998 32 97 103 10.1080/14017439850140247 9636965

[B74] Gao X Ray R Xiao Y Barker PE Ray P Inhibition of sulfur mustard-induced cytotoxicity and inflammation by the macrolide antibiotic roxithromycin in human respiratory epithelial cells BMC Cell Biol 2007 8 17 10.1186/1471-2121-8-17 17524151 PMC1890552

[B75] Urita Y Watanabe T Ota H Iwata M Sasaki Y Maeda T High prevalence of gastroesophageal reflux symptoms in patients with both acute and nonacute cough Int J Gen Med 2009 1 59 63 20428407 10.2147/ijgm.s4185 PMC2840545

[B76] Galmiche JP Zerbib F Varannes S Review article: respiratory manifestations of gastro-oesophageal reflux disease Aliment Pharmacol Ther 2008 27 449 464 10.1111/j.1365-2036.2008.03611.x 18194498

[B77] Theodoropoulos DS Ledford DK Lockey RF Pecoraro DL Rodriguez JA Johnson MC Prevalence of upper respiratory symptoms in patients with symptomatic gastroesophageal reflux disease Am J Respir Crit Care Med 2001 164 1 72 76 11435241 10.1164/ajrccm.164.1.2006002

[B78] Ghanei M Khedmat H Mardi F Hosseini A Distal esophagitis in patients with mustard-gas induced chronic cough Dis Esophagus 2006 19 4 285 288 10.1111/j.1442-2050.2006.00580.x 16866862

[B79] Amin AR Attur MG Thakker GD Patel PD Vyas PR Patel RN A novel mechanism of action of tetracyclines: effects on nitric oxide synthases Proc Natl Acad Sci USA 1996 93 14014e9 8943052 10.1073/pnas.93.24.14014 PMC19486

[B80] Weinberger B Laskin JD Sunil VR Sinko PJ Heck DE Laskin DL Sulfur mustard-induced pulmonary injury: therapeutic approaches to mitigating toxicity Pulm Pharmacol Ther 2011 24 1 92 99 10.1016/j.pupt.2010.09.004 20851203 PMC3034290

[B81] Shohrati M Shamspour N Babaei F Harandi AA Mohsenifar A Aslani J Ghanei M Evaluation of activity and phenotype of alpha1-antitrypsin in a civil population with respiratory complications following exposure to sulfur mustard 20 years ago Biomarkers 2010 15 1 47 51 10.3109/13547500903268318 19788402

[B82] Ghanei M Panahi Y Mojtahedzadeh M Khalili AR Aslani J Effect of gamma interferon on lung function of mustard gas exposed patients, after 15 years Pulm Pharmacol Ther 2006 19 2 148 153 10.1016/j.pupt.2005.07.003 16137903

[B83] Hosseini-Khalili AR Thompson J Kehoe A Hopkinson NS Khoshbaten A Soroush MR Angiotensin-converting enzyme genotype and late respiratory complications of mustard gas exposure BMC Pulm Med 2008 8 15 10.1186/1471-2466-8-15 18702808 PMC2527601

[B84] Bullman T Kang H A fifty- year mortality follow-up study of veterans exposed to low level chemical warfare agent, mustard gas Ann Epidemiol 2000 10 5 333 338 10.1016/S1047-2797(00)00060-0 10942882

[B85] Das L Vinayak M Anti-carcinogenic action of curcumin by activation of antioxidant defence system and inhibition of NF-kappaB signalling in lymphoma-bearing mice Biosci Rep 2012 32 2 161 170 [Research Support, Non-U.S. Gov’t] 10.1042/BSR20110043 21831045

[B86] Schaffer M Schaffer PM Zidan J Bar Sela G Curcuma as a functional food in the control of cancer and inflammation Curr Opin Clin Nutr Metab Care 2011 14 6 588 597 10.1097/MCO.0b013e32834bfe94 21986478

[B87] Li Y Chen ZQ Li YD Effects of curcumin on the epithelial mesenchymal transition and TGF-beta/Smads signaling pathway in unilateral ureteral obstruction rats Zhongguo Zhong Xi Yi Jie He Za Zhi 2011 31 9 1224 1228 22013801

[B88] Adelipour M Imani Fooladi AA Yazdani S Vahedi E Ghanei M Nourani MR Smad molecules expression pattern in human bronchial airway induced by sulfur mustard Iran J Allergy Asthma Immunol 2011 10 3 147 154 21891820

[B89] Zhang D Huang C Yang C Liu RJ Wang J Niu J Antifibrotic effects of curcumin are associated with overexpression of cathepsins K and L in bleomycin treated mice and human fibroblasts Respir Res 2011 12 154 [Research Support, Non-U.S. Gov’t] 10.1186/1465-9921-12-154 22126332 PMC3260240

[B90] Rennolds J Malireddy S Hassan F Tridandapani S Parinandi N Boyaka PN Curcumin regulates airway epithelial cell cytokine responses to the pollutant cadmium Biochem Biophys Res Commun 2012 417 1 256 261. 6 [Research Support, N.I.H., Extramural Research Support, Non-U.S. Gov’t] 10.1016/j.bbrc.2011.11.096 22142850 PMC3259219

[B91] Sharma RA Steward WP Gescher AJ Pharmacokinetics and pharmacodynamics of curcumin Adv Exp Med Biol. [Review] 2007 595 453 470 10.1007/978-0-387-46401-5_20 17569224

[B92] Hom J Fernandes RM When should nebulized hypertonic saline solution be used in the treatment of bronchiolitis? Paediatr Child Health 2011 16 3 157 158 22379380 10.1093/pch/16.3.157 PMC3077306

[B93] Burrows EF Southern KW Noone PG Sodium channel blockers for cystic fibrosis Cochrane Database Syst Rev 2012 3 CD005087 10.1002/14651858.CD005087.pub3 22419304

[B94] Yaghi A Zaman A Dolovich MB The Direct Effect of Hyperosmolar Agents on Ciliary Beating of Human Bronchial Epithelial Cells J Aerosol Med Pulm Drug Deliv 2012 10.1089/jamp.2011.0914 22280546

[B95] Reeves EP Williamson M O’Neill SJ Greally P McElvaney NG Nebulized hypertonic saline decreases IL-8 in sputum of patients with cystic fibrosis Am J Respir Crit Care Med 2011 183 11 1517 1523 10.1164/rccm.201101-0072OC 21330456

[B96] Al-Ansari K Sakran M Davidson BL El Sayyed R Mahjoub H Ibrahim K Nebulized 5% or 3% hypertonic or 0.9% saline for treating acute bronchiolitis in infants J Pediatr 2010 157 4 630 634 4 e1 10.1016/j.jpeds.2010.04.074 20646715

[B97] Hurt K Bilton D Inhaled mannitol for the treatment of cystic fibrosis Expert Rev Respir Med 2012 6 1 19 26 10.1586/ers.11.87 22283575

[B98] de Nijs SB Fens N Lutter R Dijkers E Krouwels FH Smids-Dierdorp BS Airway inflammation and mannitol challenge test in COPD Respir Res 2011 12 11 10.1186/1465-9921-12-11 21241520 PMC3036630

